# Generative AI Needs a Pilot: Positioning Advanced Practice Nurses as the “Human-in-the-Loop” for Primary Care Safety

**DOI:** 10.31662/jmaj.2026-0174

**Published:** 2026-07-10

**Authors:** Takeshi Miura, Tomohiko Takahashi, Kazumi Kubota

**Affiliations:** 1 Department of Gerontological Nursing, School of Medicine, Yokohama City University, Yokohama, Japan; 2 Department of Health Data Science, Yokohama City University, Yokohama, Japan; 3 University of Tsukuba Hospital, Tsukuba, Japan; 4 Doctoral Program in Medical Sciences, Graduate School of Comprehensive Human Sciences, University of Tsukuba, Tsukuba, Japan; 5 Department of Women’s Health, Population Sciences, National Center for Child Health and Development, Japan; 6 Department of Healthcare Information Management, The University of Tokyo Hospital, Tokyo, Japan

**Keywords:** generative artificial intelligence (GenAI), verification tax, human-in-the-loop (HITL), advanced practice nurses (APNs), patient safety

## Abstract

Generative artificial intelligence (GenAI) is increasingly integrated into primary care, supporting documentation, clinical summaries, and patient-facing communication. While its potential to reduce cognitive workload is widely recognized, GenAI introduces a safety paradox: as outputs become more fluent, inaccuracies such as hallucinations and omissions may become harder to detect. This creates a largely unrecognized burden in routine care, referred to as the “verification tax,” which risks redistributing rather than alleviating clinician workload.

A central question is not whether GenAI is sufficiently accurate, but how its outputs are incorporated into clinical workflows and safety structures. In this commentary, we propose a human-in-the-loop (HITL) model that positions advanced practice nurses (APNs) as first-pass reviewers for high-volume, context-sensitive GenAI outputs. This role focuses on routing, contextualization, and verification, ensuring that machine-generated content is appropriately interpreted before influencing patient care. Given their roles in patient education, care coordination, and longitudinal management, APNs may be well-suited to address the gap between standardized model outputs and individualized clinical contexts.

A conservative routing architecture and minimum governance framework are outlined, including explicit safety boundaries, structured escalation pathways, and the need for protected time for verification work. Potential limitations include the risk of redistributing invisible labor, model and workflow drift, and equity concerns in patient-facing communication. These considerations suggest that appropriate resourcing, audit mechanisms, and role-based governance are necessary.

Positioning APNs as first-pass HITL reviewers may offer a pragmatic approach to integrating GenAI into primary care while supporting patient safety and interprofessional collaboration.

## Introduction

Generative artificial intelligence (GenAI) in primary care has shifted from a futuristic possibility to an immediate operational reality. Large language models (LLMs) are already being used to draft documentation, summarize histories, and generate patient-facing text. The promise of cognitive offloading is understandably attractive for the primary care workforce facing projected shortages and rising burnout ^(1)^. However, GenAI introduces a paradox that health systems risk underestimating: the more fluent an output becomes, the harder it is to detect what is wrong.

A central safety concern is not simply that LLMs can be inaccurate but that their inaccuracies can be subtle, plausible, and therefore costly to detect. LLM-generated clinical narratives may resemble clinician-written text while still containing hallucinations and omissions ^(2)^. This creates a new, largely unpriced burden in everyday care: a “verification tax.” Unlike traditional double-checking, the verification tax is amplified by GenAI’s fluency; drafts appear finished, inviting premature trust and hiding omissions that clinically matter. If verification is implicitly assigned by default to physicians, GenAI risks becoming a workload shift rather than relief, worsening the pressure it was intended to address ^(1)^. From a patient safety and human factors perspective, this burden is not merely additional workload ^(3)^. It represents a new form of cognitive safety work created by automation bias, reduced situation awareness, and the need to detect latent errors in seemingly coherent machine-generated text. Because fluent outputs can shift clinicians from active formulation to passive verification, the verification tax may increase the risk that omissions, unsupported inferences, or context-inappropriate recommendations remain undetected until they affect clinical decisions. Framing verification as a human factors problem, therefore, clarifies why it requires explicit workflow design, protected time, and escalation rules rather than informal individual vigilance.

Therefore, the central structural question is not whether GenAI is “good enough” but “who is the pilot in an AI-augmented clinic?” In this commentary, the “pilot” is not the person holding final accountability for every decision. Rather, the pilot functions to ensure that GenAI outputs are routed, verified, contextualized, and escalated appropriately before shaping patient care (human-in-the-loop [HITL] role). This pilot function should be deliberately assigned as part of the patient safety architecture. A pragmatic and safe approach is to position advanced practice nurses (APNs) as first-pass HITL reviewers for high-volume, context-sensitive GenAI outputs in primary care.

The novelty of this commentary is threefold. First, rather than treating HITL oversight as a generic safety principle, we specify a role-based clinical workflow in which APNs serve as first-pass reviewers for high-volume, context-sensitive GenAI outputs. Second, we conceptualize the “verification tax” not merely as an individual workload issue, but as a patient safety and team-design problem that must be explicitly allocated within primary care workflows. Third, we distinguish this model from physician-centered or automation-heavy governance approaches by proposing a conservative routing architecture in which APNs verify, contextualize, and escalate GenAI outputs before physician decision-making is required for high-stakes actions. Thus, the contribution of this article is not the general claim that humans should supervise AI, but the operational specification of who should perform first-pass verification, for which types of outputs, and under what escalation boundaries in primary care.

## The Last-Mile Problem in Primary Care

In this commentary, APNs are used to refer to advanced nursing roles (e.g., nurse practitioners and clinical nurse specialists), recognizing that practice and authorization scopes vary by jurisdiction. The argument is intentionally conservative: the proposed APN pilot function focuses on communication, documentation, and protocolized low-risk triage, areas where context and safety framing dominate, while reserving high-stakes decisions, medication changes, and executable orders for authorized prescribers.

The fundamental limitation of current LLMs is not a lack of biomedical knowledge but patient-specific context. They excel at processing standardized information yet struggle with the “messy” reality of individual values, social determinants, and longitudinal narratives driving primary care decisions ^(4)^. In primary care, feasibility, preferences, function, caregiver support, and trust are not marginal, but are core clinical inputs. Hager et al. ^(4)^ emphasized that current LLMs are not ready for autonomous clinical decision-making and require systematic evaluation and mitigation. Operationally, safety must be built into the workflow and not bolted on after harm is detected.

## Why APNs Are Well-Positioned as First-Pass HITL

APNs are well-positioned to address this “last-mile” gap because nursing practice is inherently contextual and relational. In many settings, APNs perform care coordination, patient education, chronic disease management, and longitudinal monitoring, domains where GenAI generates high volumes of text and carries nuance risk. Assigning APNs as first-pass HITL reviewers creates a division of cognitive labor rather than leaving it to default. Team-based primary care is the cornerstone of workforce sustainability ^(5)^. GenAI raises the teamwork stakes because it introduces a new safety work category, validating machine-generated drafts, which would otherwise default to the most visibly accountable clinician. As workforce constraints intensify ^(1)^, leaving verification unassigned is a predictable design failure.

A conservative workflow is as follows: GenAI drafts content, the APN contextualizes and performs first-pass validation, and the physician makes the final decision for high-stakes clinical reasoning, medication changes, and executable actions.

This APN-centered model differs from both physician-centered and automation-heavy approaches. In a physician-centered model, all GenAI outputs ultimately flow to physicians for review, which may preserve accountability but risks increasing inbox burden and undermining the intended benefit of cognitive offloading. In an automation-heavy model, GenAI outputs may be routed directly to documentation or patient-facing communication with minimal human mediation, which may improve efficiency but increases the risk that fluent errors, omissions, or context-inappropriate messages reach patients or records. By contrast, the APN-centered model creates an intermediate safety layer: APNs review high-volume outputs that require contextual judgment, filter routine issues, identify red flags, and escalate only clinically consequential or high-risk matters to physicians. This structure advances current practice by converting AI oversight from an implicit physician burden into an explicit, team-based safety function.

Rather than requiring physicians to review raw GenAI output, APNs remove hallucinations, confirm chart consistency, tailor patient-facing language to literacy and culture, and flag feasibility barriers or risks that models cannot reliably capture. This approach aligns with “practice at the top of license” without overextending scope. Nursing informatics scholarship has already highlighted that GenAI integration raises educational, regulatory, and ethical questions requiring nursing leadership rather than passive adoption ^(6)^. An APN-centered HITL role is a concrete way to operationalize leadership at the point of care.

## Relation to Emerging Clinical AI Agents

This model should also be understood in relation to emerging clinical AI agents and agentic workflows. Recent work has described a shift from dialogue-based medical chatbots to workflow-embedded clinical AI agents characterized by persistent state management, tool orchestration, workflow coupling, and bounded autonomy ^(7)^. These systems may not only generate text but also retrieve information, coordinate multi-step tasks, and initiate workflow actions within institutional processes. As clinical AI becomes more agentic, the safety question therefore expands from whether a single output is accurate to whether a sequence of AI-mediated actions remains contextually appropriate, auditable, and aligned with clinical accountability.

The APN-centered HITL model proposed here is intentionally conservative in this context. It does not assume that APNs should supervise autonomous diagnosis, prescribing, or executable clinical orders. Rather, APNs function as a first-pass safety layer for high-volume, context-sensitive outputs, while actions involving medication changes, diagnosis, or executable orders remain subject to authorized clinician review. In this sense, the proposed model complements emerging agentic AI systems by defining human control points, escalation boundaries, and role-based accountability before AI-generated outputs or actions influence patient care.

## A Conservative Routing Architecture

Safety depends on a clear routing and accountability architecture: who reviews what, what triggers escalation, and what cannot be allowed to happen automatically. Figure 1. Proposed APN-centered HITL workflow for GenAI outputs in primary care. Table 1 presents a conservative triage model assigning APNs as the default HITL reviewers for patient-facing text, documentation drafts, and protocolized low-risk triage support. The model intentionally maintains hard safety boundaries: GenAI outputs are advisory, no patient-facing messages are sent without documented human review, and medication changes and executable orders require authorized prescriber review. Such conservatism is needed to mitigate LLM limitations through structured safeguards ^(4)^, as LLM-generated narratives may include hallucinations and omissions even when judged “high quality” ^(2)^.

**Figure 1.**
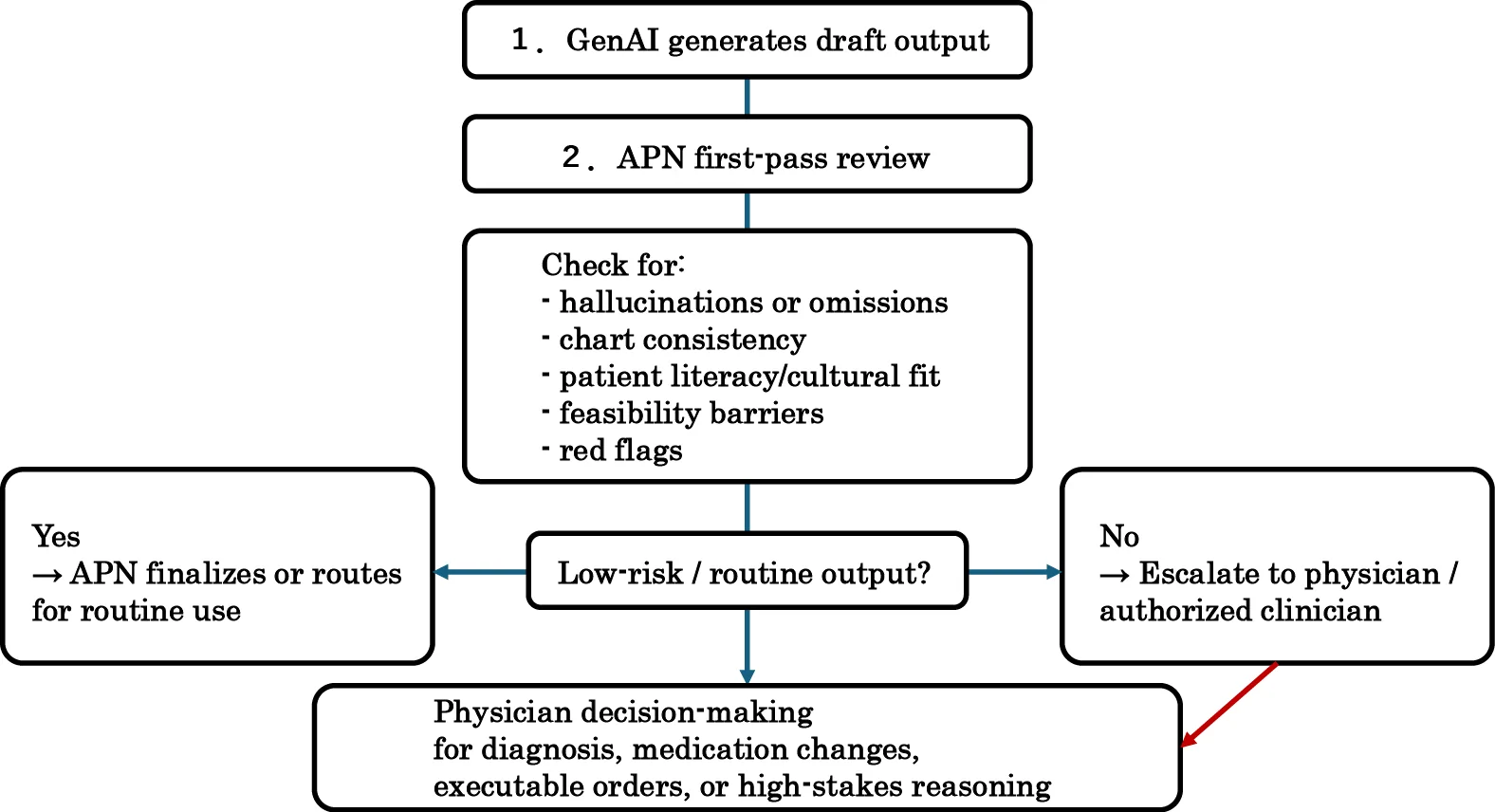
Proposed APN-centered human-in-the-loop workflow for GenAI outputs in primary care.

**Table 1.** Conservative Triage of Generative Artificial Intelligence Outputs in Primary Care: Assigning APNs as Human-in-the-Loop (HITL) for Communication, Documentation, and Protocolized Low-Risk Triage.

GenAI output (examples)	Risk if wrong	Default HITL reviewer	What the APN verifies/corrects (minimum)	Escalate to a physician when…	Safety boundary (non-negotiable)
Patient-facing text (after-visit instructions; portal replies; education materials)	Low–moderate	APN	Chart-consistency; remove hallucinations; plain-language; add red-flag precautions; avoid unintended “plan changes”	Red flags: new diagnosis/treatment implied; meds beyond refills	No message is sent without documented human review
Visit documentation drafts (HPI/SOAP; problem list suggestions)	Moderate	APN → physician sign-off	Fact-check key items; add context (function, barriers/SDOH); flag uncertainty	Diagnostic uncertainty; safety-critical omissions; conflicting history	AI drafts are not part of the legal record until a human signs
Protocolized low-risk triage support (routing; standardized pathways)	Moderate–high	APN (within protocol)	Apply protocol; confirm eligibility; screen red flags; document rationale	Outside protocol; vulnerable context; repeated contacts; any red flag	APN authority is limited to pre-specified protocols
Clinical reasoning support (differential; guideline reminders; test suggestions)	High	Physician (APN supports)	Context overlay (preferences, feasibility); identify missing data; challenge assumptions	Always a physician-owned decision	AI is advisory only
Medication-related suggestions (interactions; deprescribing prompts)	Very high	Physician/pharmacist (APN assists)	Reconcile meds/OTC/adherence; identify discrepancies	Any initiation/change	No medication changes without authorized prescriber review
Executable actions (orders, prescriptions, referrals)	Very high	Physician only	Optional: prepare drafts/check prerequisites (policy-dependent)	Always	Hard stop: AI cannot place orders; humans must authorize

Note: This model is intentionally conservative and designed to be adaptable across jurisdictions by keeping medication changes and executable actions under authorized prescribers. APN: advanced practice nurse; HPI: history of present illness; OTC: over-the-counter; SDOH: social determinants of health; SOAP: subjective objective assessment plan.

## Minimum Viable Governance for Nurse Leaders

To help nurse leaders operationalize this model, a minimum viable governance package that is feasible even in resource-constrained settings is advisable. First, “non-negotiables” in policy language are defined: (1) GenAI outputs are advisory only; (2) no patient-facing message is sent without documented human review; and (3) any medication changes or executable orders require an authorized prescriber’s review. These boundaries are set because fluent narratives may still contain clinically relevant hallucinations and omissions ^(2)^, and because current LLMs are not ready for autonomous clinical decision-making ^(4)^.

Second, routing rules in the electronic health record (EHR) are specified as a patient safety intervention rather than an IT enhancement. The simplest configuration is an APN-first queue for (a) patient-facing text and (b) documentation drafts, with physician reviews reserved for escalations and final sign-offs, consistent with the practical goal of sustaining team-based primary care under workforce pressure by designing a cognitive labor division ^(1), (5)^.

Third, verification is visible and resourced. APN review time is protected and supported by brief training on common failure modes (hallucinations, omissions, overconfidence, and escalation). Treating GenAI oversight as nursing informatics and safety competency aligns with the broader need for nursing leadership in the ethical and regulatory integration of GenAI into practice ^(6)^.

Finally, global adaptability is retained by framing the “pilot function” as role- rather than title-based. Where APN roles are limited, the first-pass review function is assigned to appropriate nursing roles (e.g., senior RNs or nurse informaticists), while prescribing remains with authorized clinicians. This maintains the safety boundaries listed in Table 1 while allowing diverse health systems to adopt the core architecture without waiting for uniform regulation.

## Policy Implications

Implementing an APN-centered HITL model requires feasible and cautious policies and management shifts. First, “supervision” should include algorithmic supervision. Regulatory and organizational language often centers on the physician’s supervision of APNs, but GenAI adds a new supervision target: machine-generated text and suggestions. Clinical governance authorizes APNs to validate and finalize high-volume, context-sensitive GenAI outputs (e.g., patient education texts and low-risk triage pathways). This formalizes the person responsible for first-pass verification, thereby preventing safety work from being invisible and inconsistently performed.

Second, EHR workflows should be reengineered for routing, escalation, and auditability. GenAI outputs should not default to the physician’s inbox; rather, they should direct outputs to APNs for first-pass validation and reserve the physicians’ attention only for high-stakes decisions and escalations. Clinics should retain a history of GenAI drafts, APN edits, and final outputs, protecting patients and operationalizing the need for mitigation and review ^(2), (4)^.

Third, interprofessional AI safety literacy should be treated as a core competency. To become pilots, APNs require training in GenAI failure modes—hallucinations, omissions, and overconfident framing—along with automation bias and red-flag escalation. Systematic mitigation, not tool optimism, is the appropriate patient safety approach ^(4)^.

## Limitations and Safeguards

This proposal has limitations that should shape implementation. First, verification work can become invisible labor. If APNs are assigned first-pass reviews without protected time or staffing support, the verification tax may be redistributed. Workforce constraints make this risk nontrivial ^(1)^. Second, model and workflow drift should be assumed. LLM behavior can change with vendor updates and local templates; sampling and audits are therefore required to keep routing rules and escalation thresholds calibrated ^(4)^. Third, equity risks are concentrated in patient-facing communication. Routing patient-facing GenAI text through APNs can be equity-protective, but only if APNs have the authority and time to adapt communication meaningfully ^(6)^. These risks argue for resourcing and governance.

## Conclusions

Empathy cannot be automated, and trust cannot be generated by an algorithm. However, many tasks that distract clinicians—such as documentation and routine messaging—can be safely automated only with a clear safety architecture. HITL oversight does not always need to be physician-led. Assigning APNs as conservative first-pass pilots for GenAI outputs in primary care is a pragmatic approach to reducing the verification tax while strengthening patient safety and nursing leadership. A practical next step is to pilot this routing architecture and refine governance based on real workflow data.

### Clinical Resources

Agency for Healthcare Research and Quality. *Patient Safety Network (PSNet).* https://psnet.ahrq.gov/

American Medical Association. *Augmented intelligence in medicine.* https://www.ama-assn.org/practice-management/digital-health/augmented-intelligence-medicine

National Institute of Standards and Technology. *AI Risk Management Framework.* https://www.nist.gov/itl/ai-risk-management-framework

The Joint Commission and Coalition for Health AI (CHAI). *Joint Commission and CHAI released initial guidance supporting responsible AI adoption across U.S. health systems.* https://www.jointcommission.org/en-us/knowledge-library/news/2025-09-jc-and-chai-release-initial-guidance-to-support-responsible-ai-adoption

World Health Organization. *Ethics and governance of artificial intelligence for health.* https://www.who.int/publications/i/item/9789240029200

## Article Information

### Acknowledgments

The authors would like to thank colleagues for their valuable discussions and insights.

### Author Contributions

Contributed equally to the writing of the manuscript: Takeshi Miura, Tomohiko Takahashi, and Kazumi Kubota.

Supervised the study and provided overall guidance and critical revisions: Kazumi Kubota.

### Conflicts of Interest

None

### Ethics Approval and Consent to Participate

Not applicable. This manuscript is an opinion article and does not involve new clinical studies of human participants or animals.

### Statement on Artificial Intelligence

In the preparation of this manuscript, the authors utilized an artificial intelligence-based language model (ChatGPT; OpenAI, San Francisco, CA, USA) to refine English phrasing and ensure structural coherence. The core concepts, clinical opinions, and final review of the content were performed entirely by the human authors, who take full responsibility for the integrity of the work.
